# Arsenic Trioxide Reactivates Proteasome-Dependent Degradation of Mutant p53 Protein in Cancer Cells in Part via Enhanced Expression of Pirh2 E3 Ligase

**DOI:** 10.1371/journal.pone.0103497

**Published:** 2014-08-12

**Authors:** Wensheng Yan, Yong-Sam Jung, Yanhong Zhang, Xinbin Chen

**Affiliations:** Comparative Oncology Laboratory, School of Medicine and Veterinary Medicine, University of California at Davis, Davis, California, United States of America; Baylor College of Medicine, United States of America

## Abstract

The p53 gene is mutated in more than 50% of human tumors. Mutant p53 exerts an oncogenic function and is often highly expressed in cancer cells due to evasion of proteasome-dependent degradation. Thus, reactivating proteasome-dependent degradation of mutant p53 protein is an attractive strategy for cancer management. Previously, we found that arsenic trioxide (ATO), a drug for acute promyelocytic leukemia, degrades mutant p53 protein through a proteasome pathway. However, it remains unclear what is the E3 ligase that targets mutant p53 for degradation. In current study, we sought to identify an E3 ligase necessary for ATO-mediated degradation of mutant p53. We found that ATO induces expression of Pirh2 E3 ligase at the transcriptional level. We also found that knockdown of Pirh2 inhibits, whereas ectopic expression of Pirh2 enhances, ATO-induced degradation of mutant p53 protein. Furthermore, we found that Pirh2 E3 ligase physically interacts with and targets mutant p53 for polyubiquitination and subsequently proteasomal degradation. Interestingly, we found that ATO cooperates with HSP90 or HDAC inhibitor to promote mutant p53 degradation and growth suppression in tumor cells. Together, these data suggest that ATO promotes mutant p53 degradation in part via induction of the Pirh2-dependent proteasome pathway.

## Introduction

Missense mutations of the p53 gene, primarily clustered within the core DNA binding domain, occur in a large fraction of human tumors [1]. These mutations produce p53 proteins with an altered sequence-specific DNA-binding activity, which cannot induce an array of target genes of wild-type p53 for tumor suppression [2]. In addition, mutant p53 proteins are found to have oncogenic activities, defined as gain of function (GOF) [3], [4].

The effects of mutant p53 on tumor development and progression are far-reaching. Compared with p53-null mice, mutant p53 knock-in mice exhibit significantly different tumor spectra and high incidence of tumor metastasis [5]–[7]. Most importantly, clinical studies have shown that a high level of mutant p53 is correlated with more aggressive tumors and poorer outcomes [8]–[10]. Furthermore, mutant p53 is clinically significant because its expression renders cells resistant to chemotherapeutic drugs [11], [12]. Apparently, gain of function of mutant p53 is partly dependent on its transcriptional activity [5], [13]–[18], and its dominant-negative activity toward the p53 family [19]–[23].

Unlike wild-type p53, mutant p53 protein is found to evade proteasome-dependent degradation [24]–[28], leading to its hyperstabilization in tumors [29]. Several mechanisms may cause mutant p53 protein to evade proteasome-dependent degradation. One possibility is that tumor-associated stress may elicit the interaction of mutant p53 with chaperone proteins, such as HSP70 and HSP90, which inactivates E3 ligases MDM2 and CHIP and consequently stabilizes mutant p53 [24]–[27]. Indeed, inhibition of HSP90 expression or activity releases MDM2 and CHIP to degrade mutant p53 [26], [30]. Another possibility is that mutant p53 is capable of forming amyloid aggregates in tumors, which are resistant to proteasomal degradation [27], [31]. The ability of mutant p53 stabilization presents a fundamental conundrum in therapeutic intervention for cancer patients with a mutant p53. Thus, effective reactivation of the proteasome-dependent degradation of mutant p53 in cancer cells has a therapeutic significance.

Recently, we found that arsenic targets mutant p53 for degradation, leading to growth suppression in solid tumor cells [32]. Arsenic is a metalloid with a substantial efficacy and moderately adverse effects in patients with acute promyelocytic leukemia, myeloma, and myelodysplastic syndromes [33]. Interestingly, we found that arsenic induces expression of wild-type p53, TAp73, and TAp63 in tumor cells [32], [34]. These activities of arsenic provide a strategy for diminishing mutant p53 dominant-negative function and other GOF activities. Although arsenic decreases the stability of mutant p53 protein through a proteasome pathway [32], the E3 ligase that targets mutant p53 for degradation remains unknown. In this study, we will address this question to facilitate the development of arsenic trioxide (ATO) as a potential anticancer drug to control tumors with mutant p53.

## Materials and Methods

### Cell Culture

Human pancreatic cancer cell line MIA PaCa-2 (containing mutant R248W) and human keratinocyte cell line HaCaT (containing mutant H179Y/R282W) were cultured as previously described [35].

### Plasmids and siRNA

Human full-length Pirh2, Pirh2-DN (an E3 ligase defective mutant), and Pirh2-ΔRING (the RING finger domain deletion mutant) were used as previously described [36]. All Pirh2 proteins were FLAG-tagged in the N terminus. FLAG-tagged ubiquitin expression vector in pcDNA3 was used as previously described [36].

Two small interfering RNAs (siRNAs) against Pirh2, 5′-CAU GCC CAA CAG ACU UGU G dTdT-3′ and 5′-GGA AGU GCA GUG CAU AAA C dTdT-3′, and two scrambled siRNAs, 5′-GCA GUG UCU CCA CGU ACU A dTdT-3′ and 5′-GGC CGA UUG UCA AAU AAU U dTdT-3′, were purchased from Dharmacon RNAi Technologies. The siRNAs were transfected into cells using SilentFect (Bio-Rad) according to the manufacturer's protocol. Cells were harvested at the indicated times after transfection for further experiments.

### Antibodies

Rabbit anti-p53(FL-393) was purchased from Santa Cruz Biotechnology Inc. Rabbit polyclonal anti-Pirh2 was purchased from Bethyl Laboratories Inc. Mouse monoclonal anti-FLAG M2 and rabbit anti-actin were purchased from Sigma.

### Reverse transcription PCR assay

Total RNA was isolated from cells using TRIzol reagent (Invitrogen). cDNA was synthesized using an Iscript™ cDNA Synthesis kit (Bio-Rad). To measure Pirh2 mRNA, RT-PCR was done with forward primer 5′-CTGCGAGCACTATGACAGAG-3′and reverse primer 5′-TTCATAGCTAGGCATAAGTTAC-3′. Actin was amplified with forward primer 5′-TCCATCATGAAGTGTGACGT-3′ and reverse primer 5′-TGATCCACATCTGCTGGAAG-3′.

### Proteosome inhibition assay

Cells were seeded for 24 h, untreated or pretreated with proteasome inhibitor MG132 (4 µM) for 2 h, and then treated with ATO for 6 h.

### Immunoprecipitation and Western blot analysis

The immunoprecipitation experiment was carried out as previously described [37]. Briefly, HaCaT and MIA PaCa-2 cells were treated with 5 or 7.5 µM ATO for 6 h. Cells were washed with phosphate-buffered saline, lysed in lysis buffer (50 mM Tris-Cl, pH 8.0, 150 mM NaCl, 1 mM EDTA, 1% Nonidet P-40, and 0.4 mM PMSF), sonicated, and clarified by centrifugation. Cell lysates (500 µg of total proteins) were incubated for 4 h at 4°C with the indicated antibodies coupled to the protein A-agarose beads (Sigma) and then washed with lysis buffer. Immunoprecipitated protein complexes and whole-cell lysates were subjected to SDS-PAGE. For each set, 5% of whole-cell lysate was used as an input control. IgG antibody was used as a negative control. Immunoblots were visualized by SuperSignal West Femto Chemiluminiscent detection reagents (Pierce).

### GST fusion protein preparation

Glutathione S-transferase (GST)-tagged Pirh2, Pirh2-ΔRING, or Pirh2-DN was expressed by pGEX-4T-3 (Amersham Pharmacia Biotech). The recombinant GST-tagged proteins were purified as described previously [37]. Briefly, the GST fusions of Pirh2, Pirh2-ΔRING, and Pirh2-DN were expressed in E. coli BL21 (DE3) (Novagene) upon induction with 0.5 mM IPTG for 4 h at 37°C. Bacterial cells were harvested and then resuspended in GST lysis buffer (200 mM Tris-Cl, pH 8.0, 0.5 M NaCl, 100 mM EDTA, 0.1% Triton X-100, and 0.4 mM PMSF). Subsequently, cell lysates were sonicated and clarified by centrifugation. GST fusion proteins were purified using glutathione-Sepharose beads (Amersham Pharmacia Biotech) according to the manufacturer's protocol.

### p53 ubiquitination assay

^35^S-labeled wild-type p53 or mutant p53 (R175H and R273H) proteins were synthesized by in vitro transcription and translation using the TNT T7 coupled reticulocyte lysate system (Promega). 5 µl (∼2×10^4^ cpm) of in vitro translated p53 were added to GST-Pirh2 (2 µg) and mixed on ice for 1 h to form GST-Pirh2-p53 complexes. The complexes were added to ubiquitination buffer (50 mM Tris-HCl, pH 7.4, 2 mM ATP, 5 mM MgCl_2_, 2 mM DTT, and 1× energy regeneration solution (ERS)), containing E1 (100 ng), E2 (200 ng), and ubiquitin (2 µg), and incubated at 30°C for 2 h. Finally, the reaction mixtures were separated on SDS-PAGE and analyzed by autoradiography. E1, E2, ERS, and ubiquitin were purchased from Boston Biochem.

### Statistics

Two-group comparisons were analyzed by two-sided Student's *t* test. *p* values were calculated, and *p*<0.05 was considered significant.

## Results

### Arsenic trioxide degrades mutant p53 protein via the proteasome-dependent pathway

**Figure 1:**
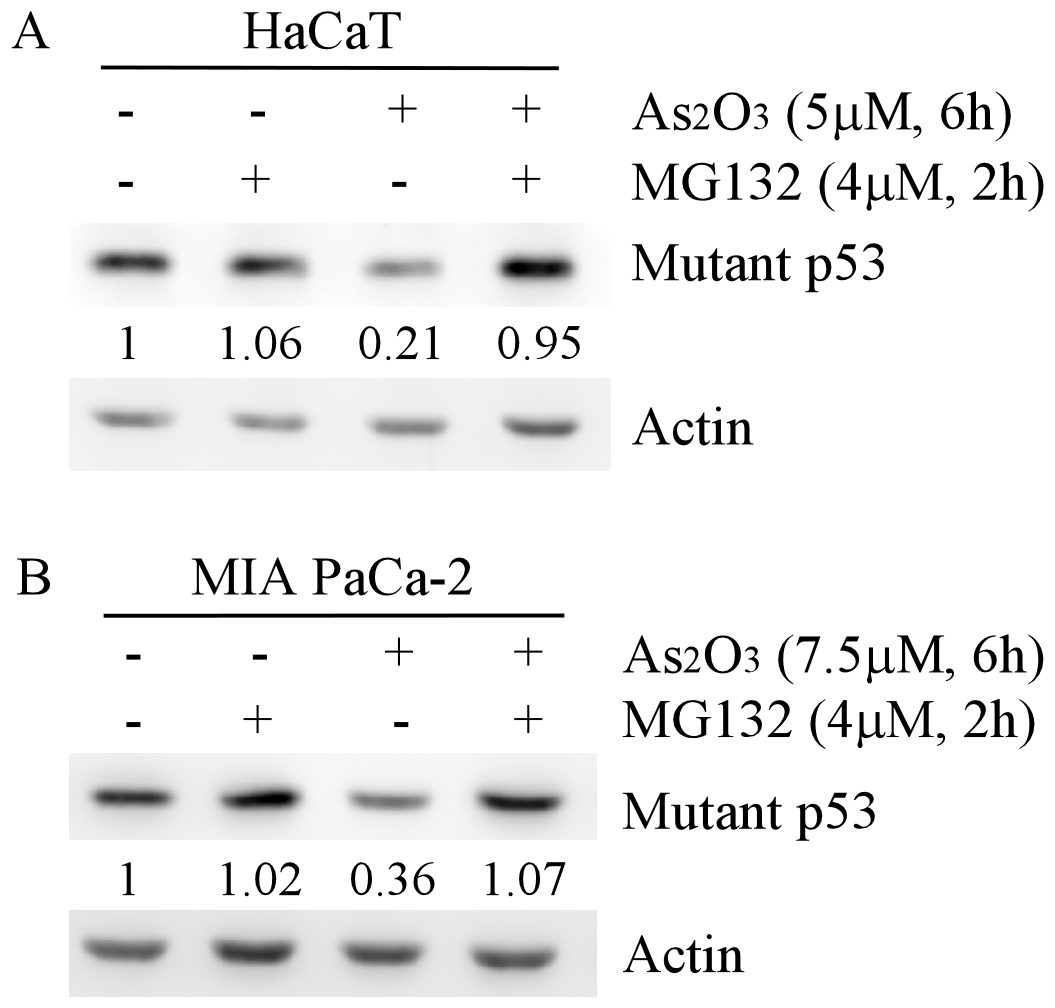
Arsenic-induced degradation of mutant p53 protein is inhibited by MG132, an inhibitor of 26S proteasome. Western blots were prepared with extracts from HaCaT (A) and MIA PaCa-2 (B) cells, which were untreated or pretreated with 4 µM MG132 for 2 h, and then untreated or treated with ATO for 6 h.

It is well-known that in tumor cells, hyperstabilization of mutant p53 protein is attributed to evasion of proteasome-dependent degradation [24]–[27], [31], [38]. Thus, reactivation of proteasome-dependent degradation of mutant p53 in tumor cells has a therapeutic significance. Previously, we found that ATO decreases the stability of mutant p53 protein through a proteasome pathway [32]. Most importantly, we showed that knockdown of endogenous mutant p53 sensitizes, whereas ectopic expression of mutant p53 desensitizes, tumor cells to arsenic treatment [32]. Consistently, another study showed that arsenic-induced degradation of mutant p53 inhibits proliferation of p53R273H-expressing cells in a dose-dependent manner [39]. However, it remains unclear which E3 ligase is responsible for arsenic-induced mutant p53 degradation. To test this, we confirmed that arsenic-induced degradation of mutant p53 is via the proteasome-dependent pathway. For this purpose, HaCaT cells were untreated or treated with 4 µM MG132, an inhibitor of 26S proteasome, in the absence or presence of ATO. We found that arsenic-induced mutant p53 degradation was completely abolished by MG132 (Fig. 1A). Similarly, we found that arsenic-induced degradation of mutant p53 protein was inhibited by MG132 in MIA PaCa2 cells (Fig. 1B). In addition, we found that, unlike the effect of the proteasome pathway on wild-type p53 protein [40], inhibition of the proteasome pathway alone was incapable of increasing the level of mutant p53 protein (Fig. 1A–B), consistent with a previous report [41]. Taken together, these results confirmed that arsenic reactivates proteasome-dependent degradation of mutant p53 in tumor cells.

### Arsenic trioxide degrades mutant p53 protein via induction of Pirh2 E3 ligase

**Figure 2:**
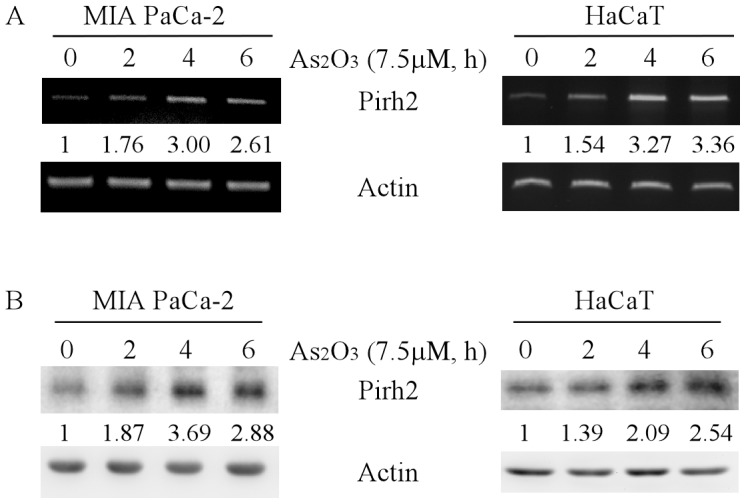
Arsenic trioxide induces expression of Pirh2 E3 ligase. (A) The level of Pirh2 transcript is increased by ATO. RT-PCR analysis was performed with total RNA isolated from MIA PaCa-2 and HaCaT cells untreated or treated with 7.5 µM ATO for 2–6 h. Actin mRNA was amplified as a loading control. (B) Western blots were prepared with extracts from MIA PaCa-2 and HaCaT cells untreated or treated as in (A), and then probed with antibodies against Pirh2 and actin, respectively.

Previously, we found that arsenic induces expression of Pirh2 E3 ligase to degrade ΔNp63 oncogenic protein, a member of the p53 family [34]. Pirh2 is known to be an E3 ligase targeting wild-type p53 protein for degradation [42], [43]. Thus, we explored whether ATO induces expression of Pirh2 in tumor cells bearing a mutant p53. We found that upon treatment with ATO, the level of Pirh2 transcripts was significantly increased in MIA PaCa-2 and HaCaT cells (Fig. 2A), concomitantly with an increase of Pirh2 protein (Fig. 2B). These data suggested that ATO may transcriptionally induce expression of Pirh2 to degrade mutant p53 in tumor cells.

**Figure 3:**
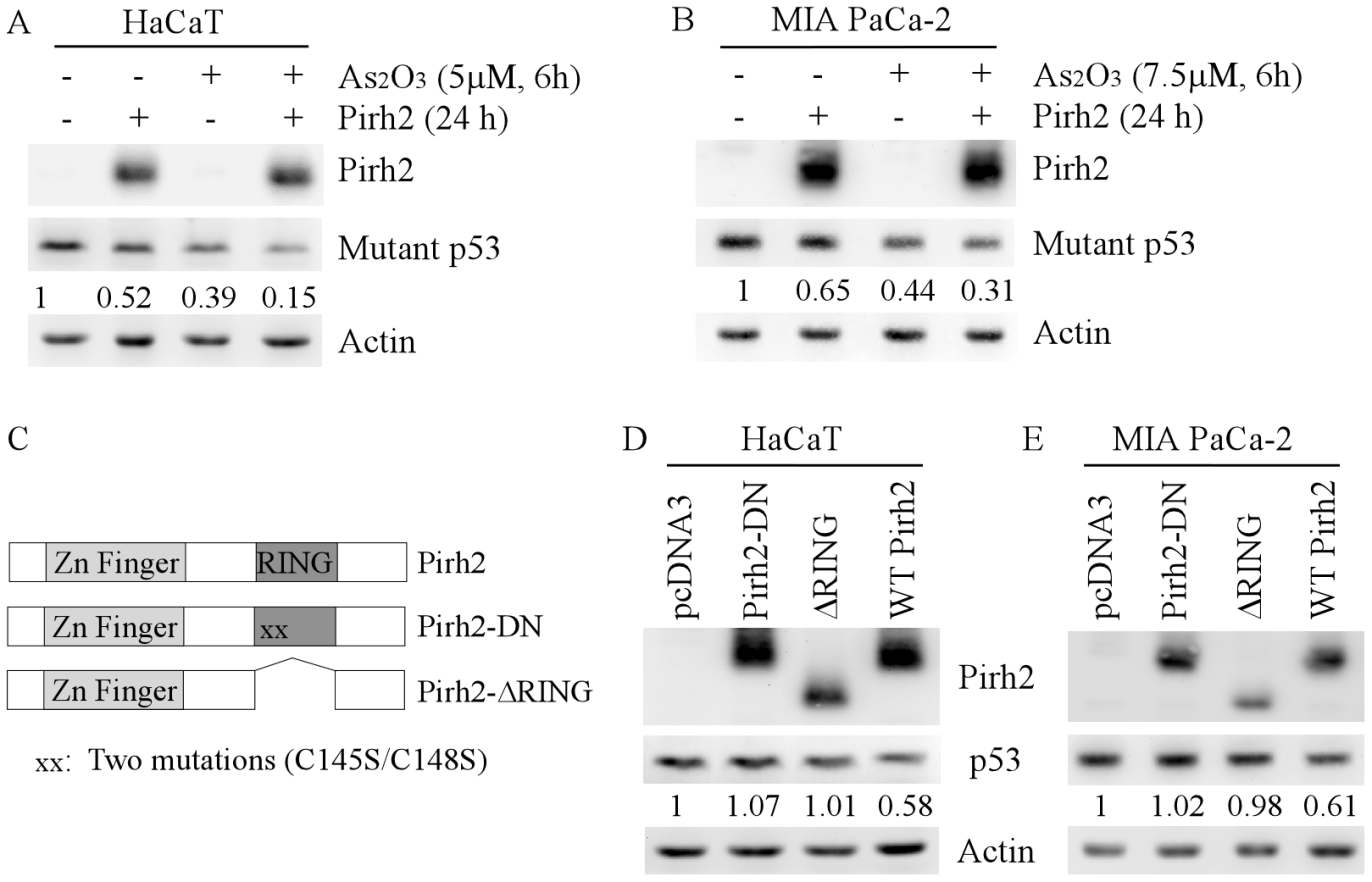
Ectopic expression of Pirh2 promotes arsenic-induced degradation of mutant p53 protein. (A–B) Western blots were prepared with extracts from HaCaT (A) and MIA PaCa-2 (B) cells, which were transfected with pcDNA3 or pcDNA3-FLAG-Pirh2 for 24 h, and then treated with 5 or 7.5 µM ATO for 6 h. The blots were then probed with antibodies against FLAG tag, p53, and actin, respectively. (C) Schematic presentation of the Pirh2 protein along with locations of the Zn Finger and RING domains, two substitution mutations C145S and C148S in the RING domain (Pirh2-DN), and deletion of the RING domain in Pirh2 (Pirh2-ΔRING). (D–E) Ectopic expression of Pirh2-DN or Pirh2-ΔRING has little if any effect on the level of mutant p53. Western blots were prepared with extracts from HaCaT (D) and MIA PaCa-2 (E) cells, which were transfected with pcDNA3, pcDNA3-FLAG-Pirh2-DN, pcDNA3-FLAG-Pirh2-ΔRING, or pcDNA3-FLAG-Pirh2 for 24 h. The blots were then probed with antibodies against FLAG tagged Pirh2, p53, and actin, respectively.

Next, we examined whether ectopic expression of Pirh2 enhances arsenic-induced degradation of mutant p53 protein. We found that ectopic expression of Pirh2 or ATO treatment alone significantly decreased the level of mutant p53 in HaCaT and MIA PaCa-2 cells (Fig. 3A–B). Most importantly, we found that a combination of ectopic expression of Pirh2 and ATO treatment further decreased the level of mutant p53 (Fig. 3A–B). These data suggested that mutant p53 can be degraded by Pirh2 E3 ligase, and the activity of Pirh2 can be enhanced by ATO treatment via unknown mechanisms.

It is known that Pirh2 requires its RING domain to ubiquitinate wild-type p53 for proteasomal degradation [44]. To determine whether E3 ligase activity of Pirh2 is required for regulating mutant p53 expression, two FLAG-tagged Pirh2 mutants, Pirh2-ΔRING (lacking RING finger domain) and Pirh2-DN (containing two substitution mutations C145S and C148S in the RING domain), were used (Fig. 3C). We showed that in contrast to wild type Pirh2, ectopic expression of Pirh2-ΔRING or Pirh2-DN was incapable of decreasing the level of mutant p53 protein in HaCaT and MIA PaCa-2 cells (Fig. 3D–E).

**Figure 4:**
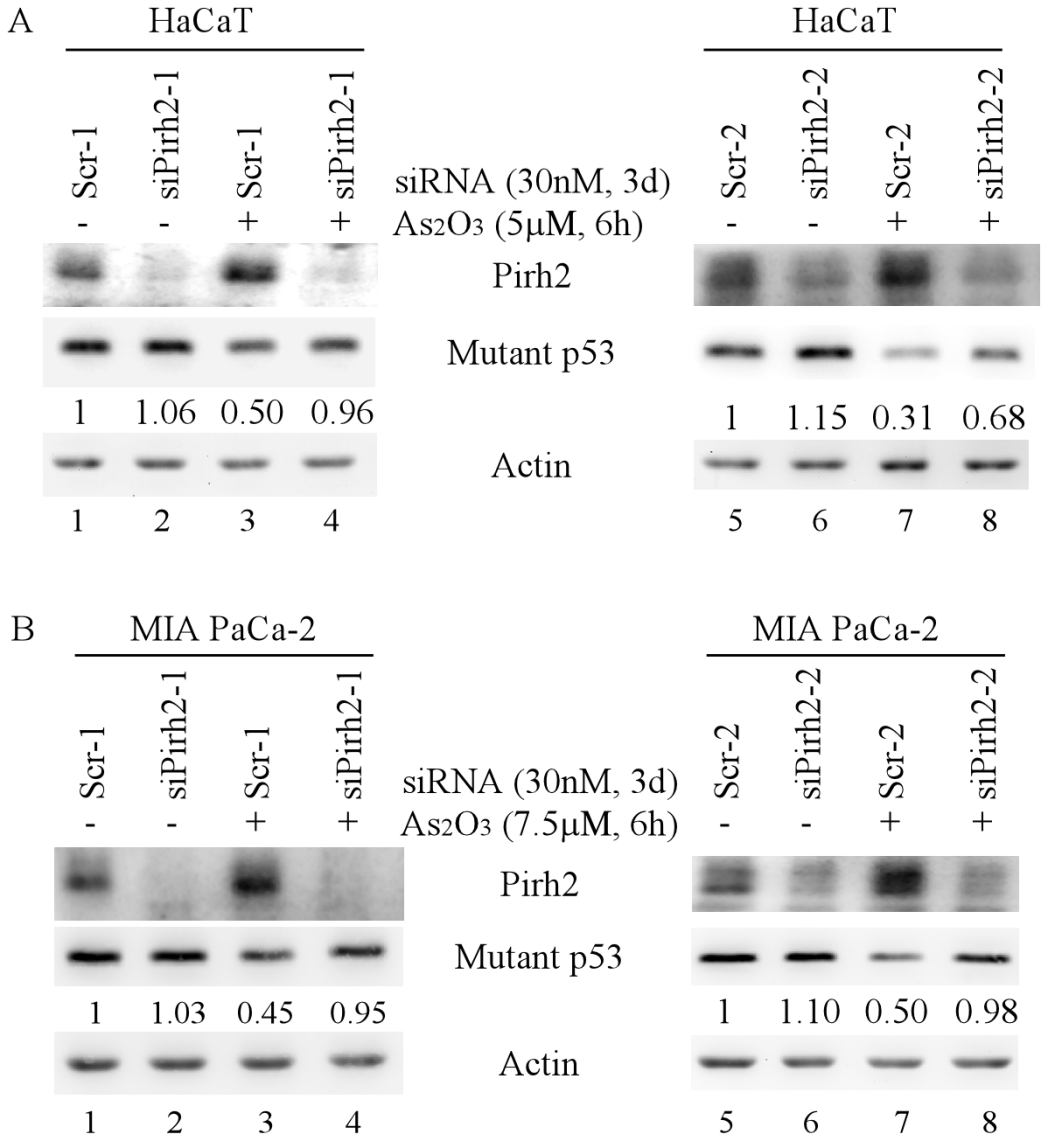
Knockdown of Pirh2 inhibits arsenic-induced degradation of mutant p53 protein. Western blots were prepared with extracts from HaCaT (A) and MIA PaCa-2 (B) cells, which were transfected with scrambled siRNA #1 (Scr-1) (lanes 1–2), Scr-2 (lanes 5–6), siRNA against Pirh2-1 (siPirh2-1) (lanes 3–4), or siPirh2-2 (lanes 7–8), and then treated with ATO for 6 h. The blots were then probed with antibodies against Pirh2, p53, and actin, respectively.

To further examine whether endogenous Pirh2 mediates arsenic-induced degradation of mutant p53 protein, HaCaT and MIA PaCa-2 cells were transiently transfected with a scrambled siRNA (Scr-1 and -2) or a siRNA against Pirh2 (siPirh2-1 and -2) for 3 d, and then mock-treated or treated with ATO. We showed that the level of Pirh2 was significantly decreased by Pirh2, but not scrambled, siRNAs (Fig. 4A–B). Importantly, we found that Pirh2 knockdown was able to rescue arsenic-induced degradation of mutant p53 (Fig. 4A–B). We also noted that arsenic treatment increased Pirh2 expression, concomitantly with a decreased expression of mutant p53 (Fig. 4A–B).

### Pirh2 physically interacts with mutant p53 protein for polyubiquitination

**Figure 5:**
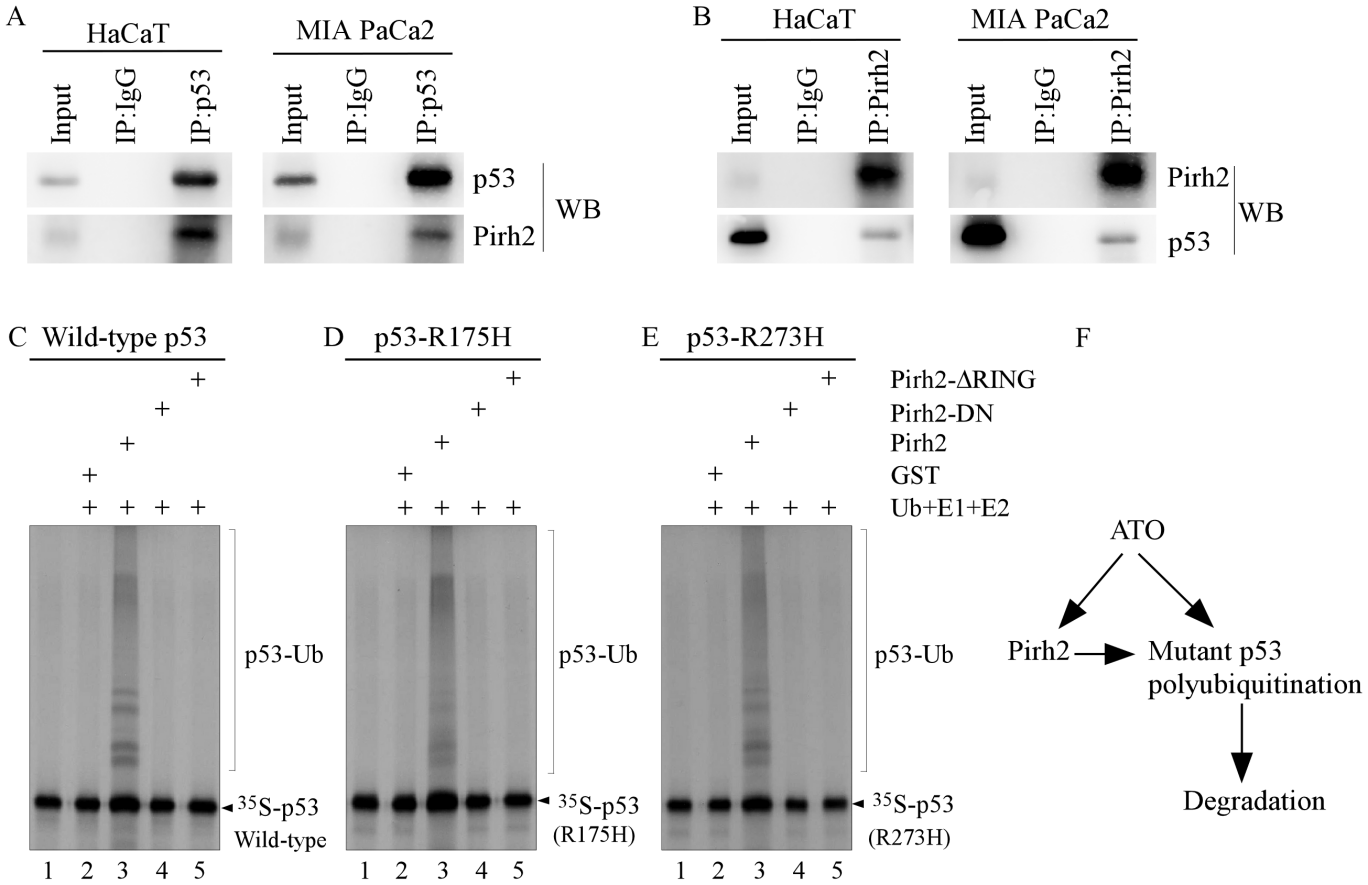
Pirh2 physically associates with mutant p53 protein for polyubiquitination. (A) HaCaT and MIA PaCa-2 cells were treated with 5 or 7.5 µM ATO for 6 h. Cell extracts from HaCaT (left) and MIA PaCa-2 (right) cells were immunoprecipitated with anti-p53 or control IgG. The immunocomplexes were then used to detect mutant p53 and Pirh2 along with whole-cell lysates as input control. (B) The experiment was performed as described in (A), except that anti-Pirh2 antibody was used in immunoprecipitation. (C–E) In vitro synthesized ^35^S-labeled wild-type p53 (C), R175H (D), and R273H (E) were mixed with GST, GST-tagged Pirh2, Pirh2-DN, or Pirh2-ΔRING. The complexes were then mixed with a buffer containing E1, E2 (UbcH5b), and Ub and then incubated at 30°C for 2 h. Ubiquitinated p53 was analyzed by SDS-PAGE and detected by autoradiography. (F) A model of Pirh2-mediated degradation of mutant p53 induced by ATO.

As E3 ligase often physically interacts with its substrates, we examined whether Pirh2 physically associates with mutant p53. To test this, HaCaT and MIA PaCa-2 cells were treated with ATO for 6 h, and then endogenous mutant p53 and Pirh2 in cells were immunoprecipitated with antibodies against p53 and Pirh2, respectively. We showed that endogenous Pirh2 was detected in mutant p53 immunocomplexes (Fig. 5A). Additionally, mutant p53 was detected in Pirh2 immunocomplexes (Fig. 5B). IgG was used as a negative control during immunoprecipitation and incapable of immunoprecipitating mutant p53 or Pirh2 (Fig. 5A–B).

Next, we investigated whether Pirh2 serves as an E3 ligase for ubiquitination of mutant p53. To test this, in vitro ubiquitination assay was performed with ^35^S-labeled mutant p53R175H or p53R273H along with recombinant GST-Pirh2, GST-Pirh2-DN, or Pirh2-ΔRING. We showed that mutant p53s were polyubiquitinated by Pirh2 but not by Pirh2-DN and Pirh2-ΔRING (Fig. 5D–E, compare lane 3 with lanes 4 and 5). Since wild-type p53 is a known target of Pirh2, wild-type p53 was used as a positive control. Similarly, we showed that wild-type p53 was polyubiquitinated by Pirh2 but not by Pirh2-DN and Pirh2-ΔRING (Fig. 5C, compare lane 3 with lanes 4 and 5). Together, these data demonstrate that Pirh2 is capable of polyubiquitinating mutant p53.

### Arsenic trioxide cooperates with HSP90 or HDAC inhibitor to promote mutant p53 degradation and growth suppression in tumor cells

**Figure 6:**
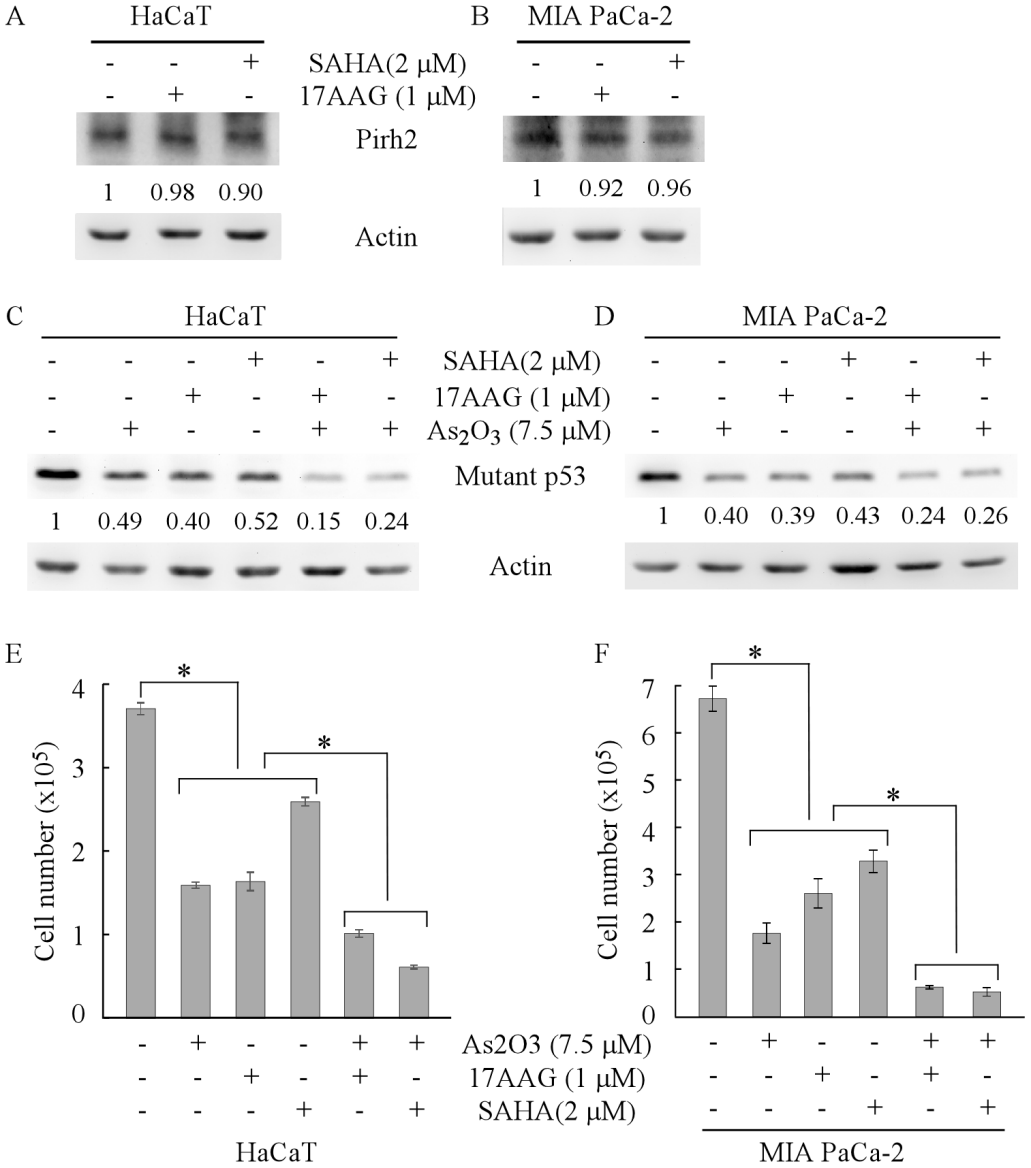
Arsenic trioxide cooperates with HSP90 or HDAC inhibitor to decrease mutant p53 expression and tumor cell proliferation. (A–B) Western blots were prepared with extracts from HaCaT (A) and MIA PaCa-2 (B) cells, which were untreated or treated with 1 µM 17AAG or 2 µM SAHA for 12 h. The blots were then probed with antibodies against Pirh2 and actin, respectively. (C–D) Western blots were prepared with extracts from HaCaT (C) and MIA PaCa-2 (D) cells, which were untreated or treated with 7.5 µM ATO, 1 µM 17AAG or 2 µM SAHA, alone or in combination for 12 h. The blots were then probed with antibodies against p53 and actin, respectively. (E–F) HaCaT (E) and MIA PaCa-2 (F) cells were treated as in (C–D) for 24 h. Surviving cells from both control and treated groups were counted and presented as Mean ± SD from three separate experiments. *, *p*<0.05.

Previous studies showed that in tumor cells, chaperone protein HSP90 interacts with mutant p53 to form MDM2-p53-HSP90 complex and consequently stabilizes mutant p53 [24], [26], [45]. Accordingly, HSP90 inhibitors 17AAG and geldanamycin disrupt MDM2-p53-HSP90 complex to degrade mutant p53 [26], [30], [45]. In addition, SAHA, an inhibitor of HDACs, was found to decrease the expression of mutant p53 via inhibiting HDAC8-mediated mutant p53 transcription [46] and HDAC6-mediated mutant p53 protein stability [30]. To further explore whether inhibitors of HSP90 and HDACs are capable of inducing Pirh2 expression to degrade mutant p53, HaCaT and MIA PaCa-2 cells were treated with 17AAG or SAHA. We showed that 17AAG or SAHA treatment had little if any effect on expression of Pirh2 protein in HaCaT (Fig. 6A) and MIA PaCa-2 cells (Fig. 6B). The result suggests that ATO and inhibitors of HSP90 and HDACs may degrade mutant p53 protein via different pathways. Thus, we hypothesized that ATO may cooperate with HSP90 or HDAC inhibitor to promote mutant p53 degradation and growth suppression in tumor cells. To test this, HaCaT and MIA PaCa-2 cells were treated with ATO, 17AAG, or SAHA, alone or in combination. We found that mutant p53 protein was markedly decreased by ATO, 17AAG, or SAHA, and further decreased by combination of ATO with 17AAG or SAHA (Fig. 6C–D). Consistently, we found that proliferation of HaCaT (Fig. 6E) and MIA PaCa-2 cells (Fig. 6F) was significantly inhibited by ATO, 17AAG, or SAHA, and further inhibited by combination of ATO with 17AAG or SAHA. These data suggest that combination of ATO with other anti-cancer agents, such as inhibitors of HSP90 and HDACs, might achieve synergistic therapies for tumors harboring a mutant p53.

## Discussion

Under normal conditions, wild-type p53 is expressed at low levels due to degradation mediated by multiple E3 ligases, including MDM2, Pirh2, COP1, ARF-BP1, and WWP1 [43]. In contrast, mutant p53 often accumulates to high levels in tumor cells, although its expression in normal tissues is also kept at low levels through the action of MDM2 [28]. The tumor-specific hyperstabilization of mutant p53 is a critical determinant of its GOF. Indeed, silencing of mutant p53 by siRNA inhibits the proliferation of human tumor cells [16], [47]. Thus, targeting mutant p53 for degradation provides a rationale for attractive anticancer strategies to attenuate the proliferation of cancer cells. Recently, it was suggested that in nonproliferating tumor cells, suppressing macroautophagy promotes the turnover of mutant p53 protein through chaperone-mediated autophagy in a lysosome-dependent manner [29]. Unfortunately, the requirement of conditional nonproliferation of tumor cells limits the potential of chaperone-mediated autophagy for clinical application. In addition, currently available chemotherapies are incapable of depleting mutant p53. For example, many frontline anticancer agents increase both wild-type p53 and mutant p53 and may promote tumor formation and progression in tumors with mutant p53 [28], [48]. Thus, stabilization of mutant p53 by chemotherapeutic agents paradoxically limits the effectiveness of these treatments.

Previously, we found that ATO, a drug clinically preferred for acute promyelocytic leukemia, decreases the stability of mutant p53 protein through a proteasome pathway, and blockage of proteasome pathway can alleviate the arsenic-induced mutant p53 degradation [32], [49]. In this study, we found that ATO induces expression of Pirh2 E3 ligase. In addition, we found that knockdown of Pirh2 inhibits, whereas ectopic expression of Pirh2 enhances, arsenic-induced degradation of mutant p53 protein. Our finding suggests that arsenic-induced expression of Pirh2 in cancer cells reactivates the proteasome-dependent mutant p53 degradation (Fig. 5F). Although mutant p53 can be targeted by MDM2 in normal cells, MDM2 cannot polyubiquitinate mutant p53 in cancer cells [28]. This defect is likely due to increased levels of HSP70 and HSP90 in cancer cells, which form complexes with mutant p53 protein [24]–[26], [45]. In addition, over-expressed MDM2 isoform B in cancer cells can interact with the full-length MDM2 and inhibit MDM2-mediated mutant p53 ubiquitination [38]. These alterations provide an opportunity to target tumors harboring a mutant p53. Indeed, we found that ATO cooperates with 17AAG or SAHA to inhibit mutant p53 expression and tumor cell proliferation.

Although ectopic expression of Pirh2 or ATO treatment alone can significantly decrease the level of mutant p53, the combination of ectopic expression of Pirh2 and ATO treatment further decreases the level of mutant p53 (Fig. 3A–B). This implies that the capability of Pirh2 E3 ligase to degrade mutant p53 can be enhanced by ATO treatment. One possibility may be due to ATO-induced posttranslational modifications of mutant p53 and Pirh2. Indeed, it was reported that upon administration of arsenic in acute promyelocytic leukaemia (APL), PML-RARα fusion oncoprotein undergoes SUMOylation by small ubiquitin-like modifiers (SUMO) and are then subjected to RNF4-mediated proteasomal degradation [50], [51]. Inactivation of SUMOylation completely blocks PML degradation [52]. Thus, further studies are warranted to investigate whether Pirh2 and/or mutant p53 are modified by SUMO, which can be enhanced by treatment of ATO in tumor cells.
